# Integrated Exhaled VOC and Clinical Biomarker Profiling for Predicting Bronchodilator Responsiveness in Asthma and COPD Patients

**DOI:** 10.3390/diagnostics15212738

**Published:** 2025-10-28

**Authors:** Malika Mustafina, Artemiy Silantyev, Aleksander Suvorov, Alexander Chernyak, Olga Suvorova, Anna Shmidt, Anastasia Gordeeva, Maria Vergun, Daria Gognieva, Sergey Avdeev, Vladimir Betelin, Philipp Kopylov

**Affiliations:** 1 Department of Cardiology, Functional and Ultrasound Diagnostics, I.M. Sechenov First Moscow State Medical University (Sechenovskiy University), 119991 Moscow, Russia; 2 Pulmonology Scientific Research Institute under Federal Medical and Biological Agency of Russian Federation, 115682 Moscow, Russia; achi2000@mail.ru (A.C.);; 3 Research Institute for Systemic Analysis of the Russian Academy of Sciences, 117218 Moscow, Russia; 4 Institute of Personalized Cardiology of The Center “Digital Biodesign and Personalized Healthcare” of Biomedical Science and Technology Park, I.M. Sechenov First Moscow State Medical University (Sechenovskiy University), 119991 Moscow, Russiasuvorov_a_yu_1@staff.sechenov.ru (A.S.);; 5 Pulmonology Department, I.M. Sechenov First Moscow State Medical University (Sechenovskiy University), 119991 Moscow, Russia; olga.a.suvorova@mail.ru (O.S.);

**Keywords:** exhaled breath analysis, volatile organic compounds, metabolomics, PTR-TOF-MS, bronchial asthma, chronic obstructive pulmonary disease, bronchodilator response, machine-learning, biomarkers

## Abstract

**Background:** Asthma and chronic obstructive pulmonary disease (COPD) are prevalent obstructive lung diseases with distinct inflammatory pathways but overlapping clinical features. Bronchodilator responsiveness (BDR) is a key diagnostic criterion, yet its metabolic determinants are poorly understood. **Objective:** This cross-sectional study investigated whether integrated profiling of exhaled volatile organic compounds (VOCs) and clinical biomarkers can differentiate BA, COPD, and health, and predict BDR. **Methods:** Exhaled breath from 160 BA patients, 128 COPD patients, and 254 healthy controls was analyzed in real-time using proton-transfer reaction time-of-flight mass spectrometry (PTR-TOF-MS) during tidal and forced expiration. Clinical assessment included spirometry, fractional exhaled nitric oxide (FeNO), blood eosinophil count, and total IgE. Machine-learning (XGBoost) was employed for feature selection and model development. **Results:** Distinct VOC signatures effectively discriminated disease groups from controls and from each other. The model for distinguishing asthma from healthy controls achieved an AUC of 0.747 during normal quiet breathing and 0.710 during forced exhale. For discriminating COPD from healthy controls, the model performance was higher, with an AUC of 0.821 for normal quiet breathing and 0.856 for forced exhale. A model integrating VOC profiles with clinical biomarkers (FeNO, eosinophils, IgE) demonstrated very high accuracy in internal validation in predicting BDR (AUC = 1.000 for tidal breathing; AUC = 0.970 for forced expiration). Specific mass spectral features (*m*/*z* 79, *m*/*z* 101) were significantly associated with a positive BDR test. **Conclusions:** This study delineates disease-specific VOC signatures and underscores the profound synergy between exhaled metabolomics and clinical immunology for identifying associations treatment response, advocating for the integration of real-time breath analysis into personalized management strategies for obstructive lung diseases.

## 1. Introduction

Asthma and chronic obstructive pulmonary disease (COPD) are among the most common chronic respiratory diseases, which together affect hundreds of millions of people worldwide and contribute significantly to global morbidity, mortality, and health care costs [1]. Although both conditions are associated with airflow limitation, their underlying pathophysiology, inflammatory profiles, and responses to treatment differ significantly [2]. Asthma is typically characterized by variable airflow limitation, airway hyperreactivity, and predominantly eosinophilic inflammation, while COPD is characterized by persistent, progressive airflow obstruction and neutrophilic inflammation, although these diseases are not mutually exclusive [3]. Despite these differences, overlapping clinical features, such as in asthma-COPD overlap syndrome (ACO), often make differential diagnosis difficult, especially in elderly patients or those with long-standing disease [4].

Spirometry testing of bronchodilator responsiveness (BDR) remains one of the most widely used functional methods for assessing the reversibility of airflow limitation, providing important diagnostic and prognostic information. The response to bronchodilators, assessed by the increase in forced expiratory volume in one second (FEV_1_) or forced vital capacity (FVC) after administration of a short-acting bronchodilator, is a key diagnostic and prognostic marker for both asthma and COPD [5]. Despite the widespread use of bronchodilator and bronchoconstrictor tests to detect bronchial hyperreactivity in clinical practice, the determination of known biomarkers—such as fractional nitric oxide content in exhaled air (FeNO) and blood eosinophil count—is becoming increasingly important, particularly when prescribing targeted therapy with monoclonal antibodies [6,7]. The search for novel biomarkers may help identify a predisposition to hyperreactivity even before the onset of clinical symptoms, as well as determine specific disease endotypes for subsequent targeted therapy.

Exhaled breath analysis is a promising non-invasive tool for respiratory disease profiling, providing real-time information on metabolic and inflammatory processes [8]. Volatile organic compounds (VOCs) in exhaled air reflect endogenous biochemical pathways, including lipid peroxidation, microbiome activity, and oxidative stress, all of which are disrupted in asthma and COPD [9]. Advanced analytical techniques such as proton transfer reaction time-of-flight mass spectrometry (PTR-TOF-MS) allow high-resolution detection of specific exhaled VOCs and identification of disease-specific signatures [10,11]. Previous studies have reported various VOC models in asthma and COPD, but only a few have investigated their usefulness in predicting BDR [12,13,14].

Combining exhaled VOC analysis with known clinical biomarkers of type 2 inflammation, such as FeNO, peripheral blood eosinophil count, and total immunoglobulin E (IgE), enables a multimodal approach that may improve diagnostic accuracy. These biomarkers are closely associated with reversible airway obstruction in asthma and ACO syndrome and may provide additional information to molecular respiratory indices. The integration of these methods may provide more accurate and non-invasive prediction of BDR, facilitating earlier and more targeted treatment. The present study was designed to investigate whether dual-mode VOC profiling, captured during normal quiet breathing and forced expiration, integrated with conventional clinical biomarkers, can accurately predict BDR in patients with BA and COPD. Our specific aims were: (1) to characterize VOC patterns that differ between patients with asthma, COPD, and healthy controls; (2) to identify key VOC and clinical predictors associated with bronchial hyperreactivity; and (3) to determine whether VOC patterns in combination with clinical biomarkers can predict bronchodilator response. We hypothesized that integrated VOC and biomarker profiling would improve diagnostic accuracy and provide new insights into the metabolic determinants of BDR. It is important to note that this study employs a cross-sectional design, which is optimal for the initial discovery and characterization of biomarker associations and was strategically chosen as an essential first step to generate robust hypotheses for future longitudinal and interventional validation.

## 2. Materials and Methods

### 2.1. Study Design and Participants

This observational cross-sectional study included patients with asthma and COPD from the outpatient and inpatient departments of the University Clinical Hospital No. 1 of Sechenovskiy University (Moscow) and controls from January 2023 to December 2024. This observational cross-sectional study was specifically chosen for this initial phase of research. The primary aim was the exploratory discovery of VOC patterns and their associations with disease status and BDR. While this design cannot establish temporality or causality, it is a critical and established first step in the biomarker development pipeline, preceding more resource-intensive longitudinal or interventional studies.

The diagnosis of asthma and COPD was established according to the criteria of the Global Initiative for Asthma 2023 and Global Initiative for Chronic Obstructive Lung Disease 2023, respectively [1,3]. The criteria for exclusion from the study were age under 18 years, pregnancy, and refusal to sign an individual informed consent. The study also included healthy control participants (aged over 18 years) without any acute or chronic bronchopulmonary diseases or pregnancy. Patients with ACO syndrome were purposefully excluded. This exclusion was necessary to establish well-defined, phenotypically distinct cohorts for this initial discovery phase, thereby minimizing diagnostic heterogeneity and allowing for a clearer identification of disease-specific VOC signatures.

**Figure 1:**
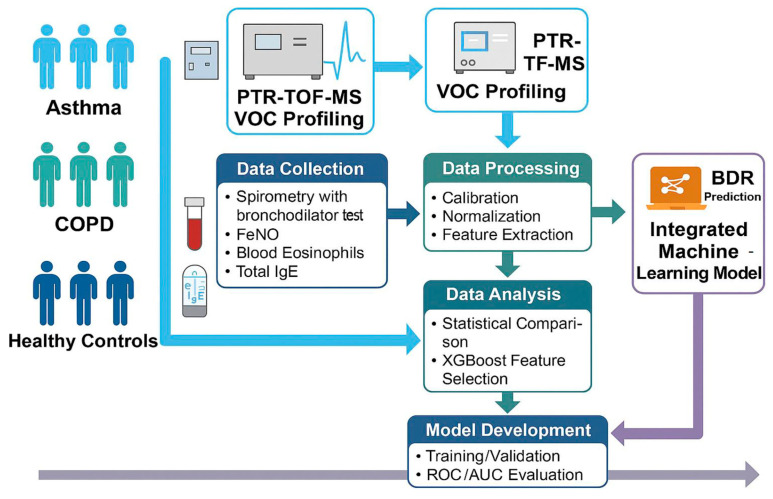
Study design scheme (AUC, Area under the curve; BDR, bronchodilator responsiveness; FeNO, fractional exhaled nitric oxide; IgE, total immunoglobulin E; PTR-TOF-MS, proton transfer reaction time-of-flight mass spectrometry; VOC, volatile organic compounds; XGBoost; eXtreme Gradient Boosting).

The study protocol was approved by the I.M. Sechenov First Moscow State Medical University Ethical Committee (Protocol No. 02-23 of 26 January 2023), and written informed consent was obtained from all study participants. The study was performed according to the Declaration of Helsinki and registered at ClinicalTrials.gov (NCT05727852). The design scheme is presented in Figure 1.

### 2.2. Collection of Clinical Data

The following clinical data were collected for each patient from medical records: age, gender, height, weight, and medications taken. Patients were asked to complete questionnaires that included demographic data such as family history, smoking history, the presence of comorbidities, and medical history. The Asthma Control Questionnaire for asthma and the Clinical COPD Questionnaire score for COPD patients were performed [15,16].

For every participant, forced spirometry and FeNO measurements were conducted in strict adherence to the joint guidelines issued by the American Thoracic Society and the European Respiratory Society [7,17,18,19,20]. The predicted values for lung function were derived from the Global Lung Function Initiative’s reference data set [17]. Forced spirometry included a BDR test, which assessed the reversibility of airway obstruction 15 min after inhalation of 400 mcg salbutamol. The magnitude of the change in FEV_1_ was used to assess the results of the bronchodilator test. A positive BDR test result was calculated using the 2022 European Respiratory Society/American Thoracic Society criteria (ΔFEV_1_ or ΔFVC > 10% predicted) [17].

The number of peripheral leukocytes, eosinophils, leukocyte formula, and total IgE in blood serum were assessed during routine outpatient and inpatient examinations.

### 2.3. Collection of Exhaled Breath and Measurement of VOCs

Exhaled breath samples were analyzed in real time using PTR-TOF-MS. Participants, including patients with asthma, COPD and healthy controls, were studied after an overnight fast and tooth brushing, always at the same time in the morning to minimize circadian and dietary effects (with the last tooth brushing and smoking occurring within 24 h prior to exhaled breath sampling). Most patients with asthma and COPD were diagnosed for the first time and were not taking baseline therapy. In the remaining patients, medications were discontinued 24 h before exhaled air sampling. Breath was drawn through a disposable mouthpiece into the Ultra-Fast PTR-TOF 1000 instrument (Ionicon, Innsbruck, Austria) equipped with a Buffered End-Tidal Breath Sampling Inlet. Mass spectra spanning *m*/*z* 10–685 were acquired at 1 s intervals using H_3_O^+^ as the reagent ion; both the drift tube and inlet were maintained at 80 °C. During a one-minute sampling period, encompassing roughly 12–16 breaths depending on respiratory rate, all spectra were recorded for downstream processing.

### 2.4. Data Processing

Raw spectral data were processed via a custom Python (v3.9) pipeline leveraging h5py, SciPy, and pandas. Every spectrum was recalibrated using three standard masses from the Ionicon PerMaSCal system (*m*/*z* 21.0220; 203.94299; 330.85). Samples exhibiting capnostat readings exceeding 3.5 units were excluded to ensure data integrity, although capnostat was not directly used to delineate breath phases due to its slower response compared to the water adduct signal ([H_2_O+H_3_O]^+^). For each spectrum, extracted ion currents (EICs) were obtained for isoprene ([M+H]^+^, *m*/*z* 69.07), dimethyl sulfide (*m*/*z* 63.02), and 1,2-butadiene (*m*/*z* 55.03), as well as the water adduct ion at *m*/*z* 37.038, which tracked moisture levels and delineated inhalation–exhalation cycles. Only spectra in which the water adduct exceeded 2 × 10^5^ cps and at least two of the three target VOCs displayed a local maximum were retained. Quality control also involved verifying calibrant mass errors (acceptable: <100 ppm, typically 10–30 ppm) and discarding samples with fewer than three full respiratory cycles. Selected spectra per sample were then summed, averaged, and smoothed using a Savitzky–Golay filter. Peaks were detected and filtered; ions present in over half of all samples were aligned across the dataset within a tolerance of ±0.015–0.4 *m*/*z*. The signals were normalized to the heavy-water isotope adduct ([D_2_O+H_3_O]^+^, *m*/*z* 22.0274) to account for potential variations in instrument sensitivity and sample humidity. This internal standard was chosen for its consistent presence in exhaled breath and its stability under the employed analytical conditions. Normalization by [D_2_O+H_3_O]^+^ provided high model accuracy in our pilot cohort (compared to normalization by the ammonia cluster [NH_4_^+^]) and was therefore chosen for this study.

### 2.5. VOCs Annotation

Significantly differing features were annotated by matching their accurate *m*/*z* values against two proprietary Ionicon libraries (300 and 1000 factory-calibrated masses) and the Human Metabolome Database (HMDB), focusing on known chronic respiratory disease markers. A mass tolerance of ±200 ppm was applied, and all identifications assumed protonation ([M+H]^+^). In addition, neutral masses were calculated as (*m*/*z*—1.00728 Da). We then exhaustively enumerated plausible elemental compositions (C, H, N, O, P, S, and, when appropriate, F, Cl, Na, Br) within ±5–10 ppm of the target mass. We also assessed whether peaks corresponded to natural isotopologues (e.g., ^13^C variants of dimethyl sulfide) by comparing relative abundance and exact-mass shifts. Characteristic fragment-ion formulas (e.g., C_3_H_5_NO^+^, C_6_H_11_^+^, C_9_H_11_^+^) were proposed based on accurate-mass data, and targeted MS/MS acquisition against HMDB, NIST, and in silico libraries (CFM-ID, MetFrag) was recommended for structural confirmation. We also consulted primary literature and computational tools (EPFL MS-Toolbox, ChemCalc) to validate typical fragmentation pathways and to support assignments of endogenous versus exogenous VOCs.

### 2.6. Statistical Data Analysis

Quantitative variables were assessed for distribution using the Shapiro–Wilk test and summarized by mean, standard deviation, median, and interquartile range. Categorical data were reported as counts and proportions. Group comparisons for normally distributed metrics employed Welch’s *t*-test (two groups) or ANOVA (more than two), with post hoc pairwise tests; non-parametric analogs (Mann–Whitney U-test, Kruskal–Wallis) were used otherwise. Categorical variables were compared via Pearson’s chi-square or Fisher’s exact test as appropriate. Statistical significance was defined at *p* = 0.05.

To identify VOCs predictive of clinical endpoints, we used a cross-validation framework combining data transformation and XGBoost. Model performance was measured by the AUC. Calibration molecules and ions with *m*/*z* < 42 were excluded.

For BA and COPD, the model was validated on a separate test set (392 for training and 150 observations for validation). For the development of diagnostic models (BA vs. controls and COPD vs. controls), the entire dataset was first stratified by the outcome variable (diagnosis) and the type of breathing maneuver (normal and forced exhale). It was then split into a training set (approximately 70% of the data) and a hold-out test set (the remaining 30%) to ensure an unbiased evaluation of model performance. This split was performed once and maintained for all subsequent analyses to prevent data leakage. The training set was used for feature selection and hyperparameter tuning of the XGBoost classifier via 5-fold stratified cross-validation. The final model, refitted on the entire training set with the selected features and optimal hyperparameters, was evaluated on the previously unseen test set to report the final performance metrics (AUC, sensitivity, specificity). The BDR model included only COPD and asthma groups and was validated with cross-validation.

For the separate analysis of normal and forced expiration, the data were divided into training and test sets. The training set was used to select the hyperparameters of the subsequent boosting classifier model. After selecting hyperparameters, features were selected separately for each type of breathing. Ranking was performed using the feature importance indicator for the boosting classifier. The top 10% of predictors by feature importance were selected.

For both models, feature selection included a resampling strategy of randomly selecting two-thirds of the training set over 1000 iterations. Each iteration included normalization and XGBoost feature-importance extraction. Median importance scores across iterations ranked VOCs; the top 30 from forced and regular breathing were compared, and overlapping VOCs were retained for further modeling.

Afterwards, the asthma and COPD XGBoost model was refitted with selected features with stratified cross-validation, and the BDR XGBoost model with selected features was refitted with leave-one-out cross-validation for both forced and tidal breathing.

Outcome probabilities were computed to derive sensitivity, specificity, positive predictive value, and negative predictive value at a 0.5 threshold.

## 3. Results

### 3.1. Baseline Characteristics

A total of 160 patients with asthma, 128 patients with COPD, and 254 healthy controls were enrolled in this observational cross-sectional study. During the selection of patients, patients with a combined diagnosis of asthma and COPD were purposefully excluded. More severe dyspnea (based on mMRC scale scores) and airway obstruction were found in patients with COPD compared with asthma (*p* < 0.001). A total of 72 (56.3%) patients with COPD had severe (GOLD III) to very severe (GOLD IV) airflow obstruction. Most smokers had a diagnosis of COPD when compared with asthma and controls (*p* ≤ 0.001). A positive BDR test, which was assessed by the change in FEV_1_ or FVC, was detected in 70.1% of patients with asthma and in 45.5% of patients with COPD. In asthma, a significant increase in FeNO, the number of eosinophils, and total IgE in the blood was noted compared to COPD (*p* ≤ 0.001). The main characteristics of all participants are presented in Table 1.

### 3.2. Identification of VOCs as Predictors of Asthma and COPD

One hundred sixty patients with asthma, 128 patients with COPD, and 254 healthy controls performed a quiet breathing maneuver, and a three-time forced expiratory maneuver, resulting in 1304 exhaled air samples. A total of 511 features in all samples were determined using PTR-TOF-MS in patients with asthma and COPD and healthy volunteers. Seventy-seven of them turned out to be significant clinical and functional predictors.

Selection using the eXtreme Gradient Boosting (XGBoost) algorithm identified the following predictors of asthma: *m*/*z* = 79.054, *m*/*z* = 95.054, *m*/*z* = 44.991 (presumably corresponds to formic acid-related fragment), *m*/*z* = 71.055, and *m*/*z* = 53.037, both in quiet breathing and forced expiratory samples. Feature importances are presented in Table 2.

**Figure 2:**
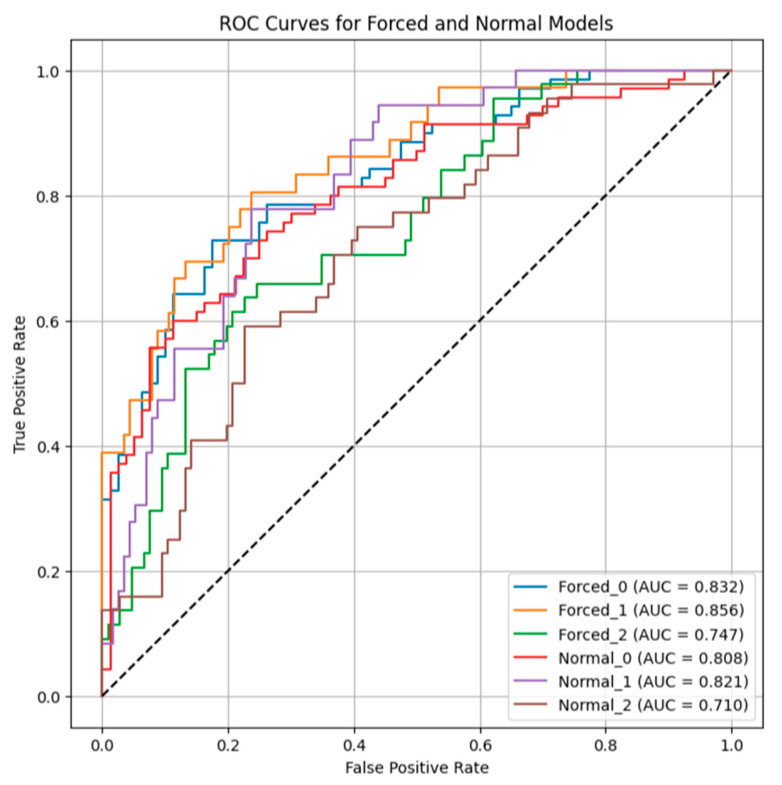
ROC curves for forced and normal expiration models in asthma, COPD, and the control group. Forced 0 (AUC = 832; sensitivity = 0.657, specificity = 0.862) and Normal 0 (AUC = 808; sensitivity = 0.614, specificity = 0.850)—ROC curves for models in healthy controls; Forced 1 (AUC = 856; sensitivity = 0.611, specificity = 0.886) and Normal 1 (AUC = 821; sensitivity = 0.556, specificity = 0.860)—ROC curves for models in COPD; Forced 2 (AUC = 747; sensitivity = 0.657, specificity = 0.862) and Normal 2 (AUC = 0.710; sensitivity = 0.659, specificity = 0.726)—ROC curves for models in asthma. AUC: Area Under the Curve; ROC—Receiver Operating Characteristic; COPD—chronic obstructive pulmonary disease.

The quality of the boosting classifier for asthma diagnosis with 11 selected VOCs was area under the ROC curve (AUC) = 0.710 (sensitivity = 0.659, specificity = 0.726) in normal quiet breathing and AUC = 0.747 (sensitivity = 0.614, specificity = 0.698) in forced exhale (Figure 2).

The same VOCs were considered COPD predictors, except for the VOC with *m*/*z* = 132.050 (detailed data are presented in Table S1). An additional predictor with *m*/*z* = 118.071 was identified for COPD: feature importance during the forced expiratory maneuver was 0.016, and for normal quiet breathing was 0.020. The quality of the boosting classifier for COPD diagnosis with 11 selected VOCs was AUC = 0.821 (sensitivity = 0.556, specificity = 0.860) in normal quiet breathing and AUC = 0.856 (sensitivity = 0.611, specificity = 0.886) in forced exhalation (Figure 2).

A detailed comparison of VOC parameters in asthma, COPD, and the control group revealed significant differences, presented in Table S2. There was a significant increase in VOCs in exhaled air with *m*/*z* = 49.005 and 95.054 in asthma compared to COPD (*p* ≤ 0.001) and a decrease with *m*/*z* = 53.037, 71.055, 77.059, 79.054, 83.086, 118.071 in asthma compared to COPD (*p* < 0.001), both in normal quiet breathing and forced expiratory maneuvers (Table S2).

### 3.3. Characterization of VOC Signatures Associated with Bronchodilator Responsiveness in Normal and Forced Expired Breath Samples

All patients with asthma and COPD underwent a bronchodilator test with salbutamol 400 mcg, which was assessed as positive if FEV_1_ or FVC ≥ 10% from baseline [17]. In patients with asthma, the average increase in FEV_1_ from baseline was 16.570 ± 14.040%, and in patients with COPD 10.583 ± 10.852% (*p* < 0.001). The characteristics of patients with BDR greater than or less than 10% are presented in Table 3.

Bronchial hyperreactivity was accompanied by a significant increase in FeNO, blood eosinophil count, total IgE, post-bronchodilator FEV_1_, FEV_1_/FVC, and FEF_75_% of predicted. In addition, in groups with a positive and negative bronchodilator response, a significant difference of 2 VOCs was noted in quiet breathing and forced expiratory samples: *m*/*z* = 79.054 and *m*/*z* = 101.039.

Selection using the XGBoost algorithm identified the following exhaled VOCs and clinical predictors of BDR: FeNO, blood eosinophil count, total IgE, pre-bronchodilator FEV_1_, post-bronchodilator FEF_25–75_% of predicted, and VOCs with *m*/*z* = 51.039, 77.059 (presumably corresponds to protonated propylene glycol), 79.054, and 101.039, both in quiet breathing and forced expiratory samples. Feature importances are presented in Table 4. ROC curves for forced and normal expiration models in BDR are presented in Figure S1. The model demonstrates high discriminatory power in the current sample, but requires validation in an independent cohort to confirm generalizability.

To address potential confounding from demographic differences between patient groups and healthy controls (specifically age, sex, and smoking status), a post hoc propensity score matching analysis was performed. The analysis yielded well-balanced cohorts for all comparisons, with no significant differences in the matched covariates (all *p* > 0.05; for detailed cohort characteristics see Supplementary Table S3).

The post hoc propensity score matching analysis confirmed the robustness of key disease-specific VOC signatures: The level of VOC *m*/*z* 79.054 remained significantly elevated in matched asthma patients compared to their demographically similar controls, both during normal quiet breathing (0.02 ± 0.022 vs. 0.015 ± 0.009, *p* = 0.018) and forced expiration (0.02 ± 0.021 vs. 0.014 ± 0.01, *p* = 0.02). The level of VOC *m*/*z* 118.071 remained significantly lower in matched COPD patients compared to their matched controls for both breathing maneuvers (normal: 0.023 ± 0.033 vs. 0.034 ± 0.041, *p* = 0.022; forced: 0.022 ± 0.034 vs. 0.030 ± 0.031, *p* = 0.028). Furthermore, the difference in VOC *m*/*z* 95.054 between asthma and COPD patients persisted in a directly matched analysis, with higher levels in the asthma group (normal: 0.189 ± 0.121 vs. 0.138 ± 0.133, *p* = 0.023; forced: 0.190 ± 0.119 vs. 0.139 ± 0.129, *p* = 0.021).

To further validate these findings, multivariate regression models were constructed, adjusting for age, sex, and smoking status (Supplementary Table S4). The results demonstrated that a diagnosis of asthma was independently associated with higher levels of *m*/*z* 79.054 (β = 0.25, *p* = 0.021) and *m*/*z* 118.071 (β = 0.31, *p* = 0.011), while a diagnosis of COPD was independently associated with higher levels of *m*/*z* 79.054 (β = 0.19, *p* = 0.038) and lower levels of *m*/*z* 118.071 (β = −0.22, *p* = 0.033) compared to the control group.

## 4. Discussion

This study presents a comprehensive analysis of exhaled VOCs in conjunction with clinical biomarkers to differentiate between asthma, COPD, and healthy controls, as well as to predict BDR. Our findings demonstrate that VOC profiling, combined with machine-learning-based feature selection, can effectively distinguish between these respiratory conditions and identify patients likely to respond to bronchodilator therapy.

### 4.1. Profile Differences and Chemical Origins of VOCs in Asthma and COPD

Our results revealed significant differences in exhaled VOC patterns between asthma, COPD, and healthy controls. Notably, VOCs with mass-to-charge ratios (*m*/*z*) 53, 71, 79, 95, and 45 (presumably corresponding to a formic acid-related fragment) were identified as key predictors of asthma and COPD, whereas *m*/*z* 118.071 was an additional discriminative marker for COPD.

Unfortunately, the annotation of ions in our study using the Ionicon libraries and the HMBD, had limitations, and the chemical origin of the ions *m*/*z* 53, 71, 79, 95 was not determined. VOC with *m*/*z* 45 probably corresponds to formic acid or acetaldehyde, related to food consumption and associated with COPD in the literature [21]. In our study, this biomarker was significantly elevated in patients with asthma and COPD compared with the control group. Probably the ion *m*/*z* 45 is a known metabolite of lipid peroxidation and is thus likely of endogenous origin, reflecting oxidative stress pathways upregulated in obstructive lung diseases. The highest content of the ion with *m*/*z* 71, according to the results of our study, was noted in patients with COPD compared to asthma and the control group (*p* < 0.001). These data are consistent with the results of the systematic review by Bos et al., where this ion was assessed as a fragment of 2-pentanone and was associated with the most common respiratory pathogens in COPD and asthma [22]. Although there is no exact match for the ion with *m*/*z* 79 in the databases (HMDB, KEGG), it is possible to assume a connection of this compound with fragments of aromatic hydrocarbons (benzene), formed as by-products of smoking [23,24]. Chemical identification of the ion with *m*/*z* 95 in a number of studies was determined differently. Thus, in the publication of Fens et al., using the same PTR-TOF-MS analytical instrument as in our study, this compound was determined as a hydroxybenzyl ion, a toluene derivative due to oxidative stress in patients with COPD [25]. In our earlier study of exhaled air in patients with cystic fibrosis, this peak has been attributed to phenol structures that correlate with bacterial infections [26,27]. For patients with COPD, one of the most significant predictors was the ion with *m*/*z* 118, and a reduced content of CO in exhaled breath was noted when compared with asthma and a group of healthy volunteers (*p* < 0.001), which is consistent with the results of Martinez-Lozano Sinues et al. [28]. In this and other studies involving patients with cystic fibrosis, this metabolite was attributed to indole derivatives and correlated with the severity of exacerbations and bacterial metabolites [27].

The XGBoost classifier demonstrated robust performance in distinguishing obstructive diseases, with AUC values of 0.747 for BA and 0.856 for COPD in forced exhalation samples. These findings align with previous studies with different analytical instruments and data processing methods suggesting that VOC signatures reflect underlying inflammatory and metabolic pathways in airway diseases [23,24,25]. The revealed differences in exhaled air (increased *m*/*z* 49 and 95 in asthma and *m*/*z* 53, 71, 77 in COPD) characterize the high discriminatory ability of these VOCs, which confirms their potential as non-invasive biomarkers for disease classification.

### 4.2. Bronchodilator Response and Predictive VOC Signatures

A critical finding of this study is the association between specific VOCs and BDR. BDR is present in a significant proportion of asthma and COPD patients, but its diagnostic utility is debated, and BDR is associated with worse lung function and higher symptom burden [29]. Analyzing the characteristics of patients in our study with a positive and negative bronchial response to a short-acting bronchodilator (Table 3), we can note statistically significant differences not only in the parameters of respiratory function, blood IgEIia, FeNO, and IgE, but also in the body mass index indicator, which can also affect the identified biomarkers in exhaled air. The higher body mass index in the BDR-positive group may reflect the known association between obesity and more severe, difficult-to-control asthma phenotypes, which often retain a significant reversible component.

However, smoking status did not differ significantly between groups (*p* = 0.065), which neutralizes the influence of this factor on the composition of exhaled air.

Earlier studies have assessed changes in exhaled air composition under the influence of inhalation therapy. Thus, in patients with COPD, bronchodilators reduced the amount of VOCs, and steroid therapy changed their composition similarly to such parameters as the BODE index and air trapping [30]. Another study assessed the change in the composition of exhaled air in asthma during inhalation therapy and its correlation with metabolites in urine [31]. In our previous study in cystic fibrosis patients, changes in VOC profiles were detected before and 20 min after inhalation of 400 μg salbutamol using real-time PTR-TOF-MS. An increase in VOCs with *m*/*z* 42 and 44 and a decrease in VOCs with *m*/*z* 71 and 89 (p˂0.001) were found in exhaled air after salbutamol inhalation [32]. However, the present study is the first to identify predictors of a positive bronchodilator response to 400 μg salbutamol. Patients with a positive BDR exhibited significantly higher levels of FeNO, blood eosinophils, and total IgE, consistent with the known role of type 2 inflammation in bronchodilator-responsive asthma [33]. Notably, two VOCs with *m*/*z* 79 and 101 were differentially expressed between BDR-positive and BDR-negative groups, suggesting their potential role in predicting therapeutic response.

Identification of significant predictors at *m*/*z* 77 and 79, along with known biomarkers (FeNO and IgE) and respiratory function indices in relation to BDR, provides new perspectives for a synergistic combination of exhaled metabolomics with traditional clinical parameters.

The prominence of *m*/*z* 77, tentatively identified as protonated propylene glycol. This metabolite in exhaled air was at its highest concentration in COPD patients when compared with asthma and a group of healthy volunteers (*p* < 0.01), which may primarily be due to exogenous sources (inhalers, smoking) [34], but may also reflect altered metabolic pathways caused by oxidative stress. This heightened oxidative stress is a cornerstone of obstructive lung disease pathology, contributing to airway Inflammation, impaired beta-2 adrenergic receptor and airway remodeling. Therefore, elevated levels of *m*/*z* 77 may reflect a state of high oxidative burden that not only drives inflammation but also directly compromises the functional integrity of the very pathway targeted by short-acting bronchodilators. This could explain its presence as a significant predictor in our model; patients with lower levels of this VOC-associated oxidative stress may have more functionally intact beta-2 adrenergic receptor signaling and thus a more robust BDR. This ion may be of exogenous origin and be a common pharmaceutical excipient and component of cigarette aerosol. The ion with *m*/*z* 51 was also a significant predictor of BDR in patients with asthma and COPD. In the study by Li et al., the ion with *m*/*z* 51 was characterized as an aryl ion of aromatic compounds and allowed reliable discrimination between patients with COPD and asthma [35]. Elevated levels of *m*/*z* 101 VOCs were noted in patients with a positive BDR during normal quiet and forced expiration (*p* < 0.03). According to the literature, the marker was associated with bacterial pathogens typical of pneumonia [35]. The presence of certain bacteria or an altered microbiome can influence airway inflammation in a way that paradoxically favors bronchodilator response.

To address the chemical interpretation of these discriminative VOCs, we have summarized the putative identities, their potential biological relevance, and supporting literature in Table 5. While the definitive identification of these ions requires future MS/MS validation, the proposed associations provide a compelling biochemical context for our findings.

### 4.3. Limitations and Clinical Implications

It is necessary to note some limitations of our study. When analyzing the data obtained, it is necessary to take into account significant differences in the factors affecting the composition of exhaled air between patients with asthma and COPD. It turned out that patients with COPD in our study were older, had a lower body mass index, were more likely to be smokers, and had more severe respiratory disorders (Table 1). To account for demographic confounders, post hoc propensity score matching and multivariate regression analyses were performed as detailed in the Supplementary Materials (Tables S3 and S4). Based on the presented data, we obtained significant differences in the groups regarding biomarkers 79 and 118; therefore, confounding factors were considered limited. The deliberate exclusion of patients with ACO syndrome, while strengthening the internal validity of our study by ensuring cohort purity, limits the immediate generalizability of our findings to this common real-world phenotype. However, to more accurately determine the predictors of obstructive diseases, additional studies are needed involving a large number of patients with asthma, COPD, and overlap syndrome, with similar baseline anthropometric characteristics, smoking status, and severity of respiratory disorders. In addition, when comparing the results of exhaled air analysis and clinical characteristics of our patients, the number of exacerbations per year and the duration of basic inhalation therapy were not taken into account. Most patients were newly diagnosed and had not previously received therapy, and if they were receiving basic therapy, it was discontinued 24 h before sampling. Known confounding factors such as smoking, eating, and brushing teeth were controlled by standardization of sampling (see Section 2.3) and we expect that their influence on the results was minimized.

The lack of structural verification by MS/MS is another limitation of the study, and future work should be aimed at accurate identification. Despite the difficulties in accurately annotating isobaric compounds or unresolved ion peaks, the use of PTR-TOF-MS allowed the detection of exhaled VOCs with sufficiently high resolution, improving diagnostic accuracy. As noted earlier, the cross-sectional design is one of the limitations of this study, preventing any causal interpretation of the observed associations between VOCs, biomarkers, and BDR. Future longitudinal studies tracking VOC profiles and BDR over time, and interventional studies, are essential to confirm whether these VOC signatures are indeed predictive of treatment response and to elucidate their causal pathways. Additionally, while the machine-learning approach optimized feature selection, external validation in independent cohorts is necessary to confirm generalizability.

From a clinical perspective, integrating VOC profiling with standard spirometry and biomarker testing could refine personalized treatment strategies. For instance, patients with high *m*/*z* 79 and 101 levels may benefit from early bronchodilator or anti-inflammatory therapy, whereas those with COPD-predominant VOC patterns might require alternative management approaches. Future studies should focus on validating these biomarkers in independent, multi-center cohorts that include patients with asthma-COPD overlap to determine the generalizability and robustness of the identified VOC signatures across the entire obstructive lung disease spectrum.

## 5. Conclusions

This study demonstrates that exhaled VOC profiling, combined with clinical biomarkers, can effectively differentiate asthma and COPD from controls and predict BDR. The identified VOC signatures provide novel insights into disease-specific metabolic perturbations and offer a promising non-invasive tool for precision medicine in respiratory disorders. Future research should focus on elucidating the biochemical pathways underlying these VOC alterations and validating their utility in larger, multi-center cohorts.

## Figures and Tables

**Table 1:** Participants’ characteristics.

	BA	COPD	Controls	*p*-Value		
				BA vs. COPD	BA vs. Controls	COPD vs. Controls
Number of subjects	160	128	254			
Gender, % male	56 (35.0%)	111 (86.7%)	98 (38.6%)	0.001	0.547	0.001
Age at enrollment, years	58.413 ± 17.055	66.609 ± 10.345	42.386 ± 18.100	<0.001	<0.001	<0.001
BMI kg·m^2^	29.294 ± 8.460	25.861 ± 5.777	26.154 ± 5.949	0.001	<0.001	0.967
Smoking status						
Never	85 (53%)	25 (19.5%)	135 (53.1%)	<0.001	<0.001	<0.001
Former	54 (33.9%)	43 (33.6%)	105 (41.3)	0.002	0.001	<0.001
Current	21 (13.1%)	60 (46.9%)	14 (5.6%)	0.001	0.007	0.001
mMRC, scores	2.054 ± 0.917	2.585 ± 0.917	0	0.001	<0.001	<0.001
FVC % pred	78.074 ± 18.664	72.647 ± 22.309	99.339 ± 11.641	0.021	<0.001	<0.001
FEV_1_% pred	61.942 ± 17.079	49.675 ± 21.627	99.614 ± 11.358	<0.001	<0.001	<0.001
FEV_1_/FVC, %	62.434 ± 9.992	51.060 ± 11.670	81.820 ± 6.197	<0.001	<0.001	<0.001
FEF_75_%pred	61.835 ± 27.019	52.499 ± 27.000	124.607 ± 53.110	<0.001	<0.001	<0.001
FVC post BD % pred	84.945 ± 17.911	77.550 ± 22.873	NA	0.006	NA	NA
FEV_1_ post BD % pred	72.298 ± 19.310	55.648 ± 24.040	NA	<0.001	NA	NA
FEV_1_/FVC post BD, %	66.305 ± 11.792	52.713 ± 13.632	NA	<0.001	NA	NA
FEF_75_ post BD %pred	78.502 ± 33.912	60.980 ± 28.523	NA	<0.001	NA	NA
BDR, %	16.570 ± 14.040	10.583 ± 10.852	NA	<0.001	NA	NA
BDR ≥ 10%	101 (70.1%)	50 (45.5%)	NA	0.001	NA	NA
FeNO, ppb	30.310 ± 23.050	24.015 ± 21.476	NA	<0.001	NA	NA
Blood eosinophils (×10^9^/L)	0.502 ± 0.428	0.407 ± 0.377	NA	0.001	NA	NA
Total IgE, IU/mL	180.321 ± 144.691	111.000 ± 124.532	NA	<0.001	NA	NA
ACQ	2.7 ± 1.1	NA	NA	NA	NA	NA
CCQ	NA	3.8 ± 4.3	NA	NA	NA	NA
GOLD class I/II/III/IV (%)	NA	10.9/32.8/37.5/18.8	NA			

Data are presented as mean ± SD or number (%). ACQ, Asthma Control Questionnaire; BA: bronchial asthma; BDR: bronchodilator responsiveness test; BMI: body mass index; CCQ: Clinical COPD Questionnaire score; COPD: chronic obstructive pulmonary disease; FeNO, fractional exhaled nitric oxide; FEV_1_: forced expiratory volume in 1 s, FVC: forced vital capacity, FEF_75_: the forced expiratory flow when 75% of FVC has been exhaled; GOLD: Global Initiative for Chronic Obstructive Lung Disease; IgE, immunoglobulin E; mMRC: Modified Medical Research Council; NA: not available; post BD: post bronchodilator value; SD: standard deviation.

**Table 2:** Predictors of asthma diagnosis by the XGBoost algorithm.

m/z	Feature Importances	
	Forced Expiratory Maneuver	Normal Quiet Breathing
44.991 *	**0.01397864**	**0.01173620**
45.992	0.00943758	0.00058650
49.005	0.00643448	0.00270099
51.039	0.00576903	0.00316140
**53.037**	**0.00494756**	**0.01042149**
69.073	0.00188930	0.00162506
**71.055**	**0.01875065**	**0.00823190**
**79.054**	**0.04068980**	**0.03198569**
83.086	0.00348485	0.00807589
**95.054**	**0.01917045**	**0.01707253**
132.050	0.00844352	0.00627082

* *m*/*z* = 44.991 presumably corresponds to the formic acid-related fragment, 141 ppm mass error (the putative chemical was identified using Ionicon libraries, the Human Metabolome Database and literature data). *m*/*z*—mass-to-charge ratio. The 5 most significant predictors are highlighted in bold.

**Table 3:** Characteristics of patients with positive and negative bronchodilator test.

Parameters	BDR ≥ 10%	BDR < 10%	*p*-Value
Number of subjects	151	103	
BA	101 (66.9%)	48 (46.7%)	0.320
COPD	55 (53.3%)	50 (33.1%)	0.450
Sex, % male	86 (57.0%)	65 (63.1%)	0.818
Age at enrollment, years	61.358 ± 15.966	62.553 ± 14.593	0.730
**BMI kg·m^2^**	**28.884 ± 8.601**	**26.549 ± 5.967**	**0.043**
Smoking status			
Never	51 (33.7%)	32 (31.0%)	0.065
Former	65 (43.0%)	35 (34.0%)	0.077
Current	35 (23.3%)	36 (35.0%)	0.065
mMRC, scores	2.183 ± 0.871	2.429 ± 1.037	0.075
FVC % pred	74.128 ± 18.769	77.765 ± 22.571	0.179
FEV_1_% pred	56.932 ± 18.179	57.783 ± 23.173	0.621
FEV_1_/FVC, %	59.398 ± 11.822	56.346 ± 12.464	0.161
FEF_75_%pred	59.830 ± 25.017	56.327 ± 28.002	0.079
FEF_25–75_% pred	77.149 ± 41.083	76.606 ± 35.248	0.121
FVC post-BD % pred	83.653 ± 18.520	79.251 ± 22.871	0.090
**FEV_1_ post-BD % pred**	**68.857 ± 20.905**	**59.923 ± 24.903**	**0.006**
**FEV_1_/FVC post-BD, %**	**63.068 ± 13.593**	**56.632 ± 14.600**	**0.001**
**FEF_25–75_ post-BD % pred**	**54.228 ± 40.606**	**40.471 ± 22.021**	**0.001**
**FEF_75_ post-BD %pred**	**77.103 ± 32.589**	**62.415 ± 31.470**	**<0.000**
**BDR, %**	**21.464 ± 11.270**	**3.002 ± 5.721**	**<0.000**
**FeNO, ppb**	**38.773 ± 23.976**	**13.878 ± 11.161**	**<0.000**
**Blood eosinophils (×10^9^/L)**	**0.654 ± 0.417**	**0.202 ± 0.220**	**<0.000**
**Total IgE, IU/mL**	**156.080 ± 121.275**	**102.248 ± 27.141**	**<0.000**
VOCs in normal quiet breathing *			
51.039	0.070 ± 0.050	0.063 ± 0.043	0.393
77.059 **	0.028 ± 0.015	0.031 ± 0.037	0.450
**79.054**	**0.025 ± 0.031**	**0.029 ± 0.026**	**0.042**
**101.039**	**0.020 ± 0.009**	**0.019 ± 0.011**	**0.024**
VOCs in forced expiratory maneuver *			
51.039	0.062 ± 0.044	0.057 ± 0.039	0.502
77.059 **	0.027 ± 0.013	0.032 ± 0.056	0.399
79.054	0.026 ± 0.030	0.028 ± 0.025	0.052
**101.039**	**0.022 ± 0.013**	**0.019 ± 0.011**	**0.003**

Data are presented as mean ± SD or number (%). BA: bronchial asthma; BDR: bronchodilator responsiveness test; BMI: body mass index; COPD: chronic obstructive pulmonary disease; FeNO, fractional exhaled nitric oxide; FEV_1_: forced expiratory volume in 1 s, FVC: forced vital capacity; FEF_25–75_: forced expiratory flow between 25% and 75% of the FVC; FEF_75_: the forced expiratory flow when 75% of FVC has been exhaled; IgE: immunoglobulin E; mMRC: Modified Medical Research Council; post-BD: post-bronchodilator value; SD: standard deviation. The most significant differences are highlighted in bold. * mass per charge value (*m*/*z*). ** *m*/*z* = 77.059 presumably corresponds to protonated propylene glycol (the putative chemical was identified using Ionicon libraries, the Human Metabolome Database and literature data).

**Table 4:** Predictors of BDR by the XGBoost algorithm.

Clinical Predictors and VOCs *	Feature Importances	
	Forced Expiratory Maneuver	Normal Quiet Breathing
Blood eosinophils, %	0.01574812	0.02867968
**FEV_1_, l**	**0.01776611**	**0.04279317**
FEV_25–75_ post-BD, l	0.01593777	0.04099168
**FEV_25–75_ post-BD, % pred.**	**0.03839435**	**0.03620051**
FEV_75_ post BD, l	0.01200586	0.01911791
**Total IgE, IU/mL**	**0.05262020**	**0.05668990**
FeNO, ppb	0.01156907	0.03228240
51.039	0.02341965	0.01837680
77.059 **	**0.04816510**	**0.03026989**
**79.054**	**0.05094414**	**0.04666028**
101.039	0.01853833	0.01798187

* VOCs data are presented as mass to charge (*m*/*z*); ** *m*/*z* = 77.059 presumably corresponds to protonated propylene glycol (the putative chemical was identified using Ionicon libraries, the Human Metabolome Database and literature data). BDR: bronchodilator responsiveness test; BMI: body mass index; COPD: chronic obstructive pulmonary disease; FeNO, fractional exhaled nitric oxide; FEV_1_: forced expiratory volume in 1 s; FVC: forced vital capacity; FEF_25–75_: forced expiratory flow between 25% and 75% of the FVC; FEF75: the forced expiratory flow when 75% of FVC has been exhaled; mMRC: Modified Medical Research Council; post-BD: post-bronchodilator value. The 5 most significant predictors are highlighted in bold.

**Table 5:** Summary of key VOCs and their putative identities and relevance.

*m*/*z* *	Putative Chemical Identity	Biological Relevance/Origin	Association in This Study	Supporting Literature
44.991	Formic acid fragment/Acetaldehyde	Product of lipid peroxidation and oxidative stress; can be influenced by diet.	Elevated in asthma and COPD vs. controls.	[21]
51.039	Aryl ion of aromatic compounds	Positive effect on mucociliary clearance, respectively, a compensatory increase in this metabolite in case of impaired clearance	Significant predictor of BDR	[35]
53.037	Not confidently identified (Potential alkyne or diene fragment)	Unknown endogenous pathway.	Decreased in asthma vs. COPD.	-
71.055	2-Pentanone, Fragments of C5-compounds	Associated with metabolic activity of common respiratory pathogens (e.g., *Pseudomonas*, *Haemophilus*); product of lipid peroxidation.	Highest in COPD vs. asthma and controls.	[22]
77.059	Protonated Propylene Glycol	Common excipient in inhalers; can also be a product of oxidative stress.	Significant predictor of BDR; highest in COPD.	[34]
79.054	Benzene/Pyridine fragment	By-product of smoking; associated with aromatic hydrocarbon exposure and oxidative stress.	Key predictor for asthma and BDR; decreased in BA vs. COPD.	[23]
95.054	Phenol/Hydroxybenzyl ion (toluene derivative)	Marker of oxidative stress; associated with bacterial infection and inflammation.	Increased in asthma vs. COPD.	[25,27]
101.039	Not confidently identified (e.g., C_6_H_12_O_2_)	In the literature, associated with bacterial pathogens (e.g., *Streptococcus pneumoniae*).	Elevated in patients with positive BDR.	[36]
118.071	Indole/Methyl Indole derivatives	Metabolites associated with bacterial activity (e.g., *Pseudomonas aeruginosa*); linked to exacerbation severity.	Additional discriminative marker for COPD.	[28]

* VOCs data are presented as mass to charge (*m*/*z*); BA: bronchial asthma; BDR: bronchodilator responsiveness test; COPD: chronic obstructive pulmonary disease.

## Data Availability

The original contributions presented in this study are included in the article/Supplementary Materials. Further inquiries can be directed to the corresponding author.

## Supplementary Materials

The following supporting information can be downloaded at: https://www.mdpi.com/article/10.3390/diagnostics15212738/s1, Table S1: Predictors of COPD diagnosis by the XGBoost algorithm; Table S2: Comparison differences in the presence of VOCs in asthma vs. COPD vs. controls; Table S3: Propensity-Matched Cohort Characteristics; Table S4: Results of Multivariate Regression Models; Figure S1: ROC curves for forced and normal expiration models in bronchodilator responsiveness.

## Abbreviations

The following abbreviations are used in this manuscript:

BA	Bronchial asthma
COPD	Chronic obstructive pulmonary disease
BDR	Bronchodilator responsiveness
PTR-TOF-MS	Proton-transfer reaction time-of-flight mass spectrometry
eVOCs	Exhaled volatile organic compounds
ATS	the American Thoracic Society
ERS	the European Respiratory Society
GLI	the Global Lung Function Initiative
AUC	Area under the curve
BMI	Body mass index
FEV_1_	Forced expiratory volume in 1 s
FEF_75_	Forced expiratory flow when 75% of FVC has been exhaled
mMRC	Modified Medical Research Council
