# Supplementary material for: Integrated Exhaled VOC and Clinical Biomarker Profiling for Predicting Bronchodilator Responsiveness in Asthma and COPD Patients
Source: Diagnostics (Basel). 2025 Oct 28;15(21):2738. doi: 10.3390/diagnostics15212738 (PMC12610987; doi:10.3390/diagnostics15212738)
Supplement: Supplementary file 1 [file diagnostics-15-02738-s001.zip › diagnostics-3914224-supplementary.pdf]

Integrated Exhaled VOC and Clinical Biomarker Profiling for Predicting Bronchodilator Responsiveness in Asthma and COPD Patients (*Supplementary Material*)

Malika Mustafina [1,2,3*], Artemiy Silantyev [4], Alexander Suvorov [4], Alexander Chernyak [2], Olga Suvorova [5], Anna Shmidt [5], Anastasia Gordeeva [5], Maria Vergun [4], Daria Gognieva [1,3,4], Sergey Avdeev [5,2], Vladimir Betelin [3], Philipp Kopylov [4,1,3]

[1] *Department Department of Cardiology, Functional and Ultrasound Diagnostics, I.M. Sechenov First Moscow State Medical University (Sechenovskiy University), 119991 Moscow, Russia*
[2] *Pulmonology Research Institute under Federal Medical and Biological Agency of Russia, 115682 Moscow, Russia*
[3] *Research Institute for Systemic Analysis of the Russian Academy of Sciences, 117218 Moscow, Russia*
[4] *Institute of Personalized Cardiology of The Center "Digital Biodesign and Personalized Healthcare" of Biomedical Science and Technology Park, I.M. Sechenov First Moscow State Medical University (Sechenovskiy University), 119991 Moscow, Russia*
[5] *Pulmonology Department, I.M. Sechenov First Moscow State Medical University (Sechenovskiy University), 119991 Moscow, Russia*

* Corresponding author. E-mail address: mustafina_m_kh@staff.sechenov.ru (Malika Mustafina)

Table S1. Predictors of COPD diagnosis by the XGBoost algorithm

m/z	Feature importances	
	Forced expiratory maneuver	Normal quiet breathing
**44.991 (formic acid-related fragment, 141 ppm mass error)**	**0.01836751**	**0.01712546**
45.992	0.00745698	0.00687134
49.005	0.00654398	0.00251098
51.039	0.00548921	0.00497851
**53.037**	**0.02967512**	**0.02398321**
69.073	0.00197865	0.00117569
**71.055**	**0.01987467**	**0.00978561**
**79.054**	**0.05791265**	**0.04872457**
83.086	0.00279123	0.00675192
**95.054**	**0.01248166**	**0.01867554**
**118.071**	**0.01636751**	**0.02044372**

*mass error is outside the range but close to 200 ppm
m/z – mass-to-charge ratio. The 5 most significant predictors are highlighted in bold

Table S2. Comparison differences in the presence of VOCs in asthma vs. COPD vs. controls*

m/z	44.991**	45.992	49.005	51.039	53.037	69.073	71.055	77.059***	79.054	83.086	95.054	101.039	118.071	132.050
**Normal quiet breathing**														
BA	2.563 ± 0.925	0.064 ± 0.053	0.036 ± 0.050	0.067 ± 0.045	0.012 ± 0.005	2.124 ± 0.932	0.160 ± 0.404	0.026 ± 0.012	0.019 ± 0.024	0.038 ± 0.113	0.194 ± 0.154	0.018 ± 0.009	0.040 ± 0.048	0.004 ± 0.004
COPD	2.531 ± 1.060	0.072 ± 0.075	0.020 ± 0.028	0.065 ± 0.046	0.014 ± 0.006	2.281 ± 0.963	0.377 ± 0.694	0.034 ± 0.036	0.037 ± 0.031	0.048 ± 0.044	0.141 ± 0.140	0.020 ± 0.011	0.023 ± 0.036	0.003 ± 0.003
Controls	2.227 ± 0.676	0.046 ± 0.024	0.026 ± 0.034	0.090 ± 0.052	0.010 ± 0.004	1.907 ± 0.812	0.074 ± 0.082	0.024 ± 0.012	0.013 ± 0.010	0.044 ± 0.202	0.211 ± 0.130	0.014 ± 0.006	0.040 ± 0.038	0.003 ± 0.003
P value***	<0.000	<0.000	<0.000	<0.000	<0.000	<0.000	<0.000	0.001	<0.000	<0.000	<0.000	<0.000	<0.000	0.008
BA vs. controls	<0.000	<0.000	0.037	<0.000	<0.000	0.014	<0.000	0.435	0.002	<0.000	0.033	<0.000	0.152	0.006
COPD vs. controls	<0.000	<0.000	0.028	<0.000	<0.000	<0.000	<0.000	0.001	<0.000	<0.000	<0.000	<0.000	<0.000	0.809
BA vs. COPD	0.824	0.558	<0.000	0.906	0.002	0.275	<0.000	0.022	<0.000	<0.000	0.001	0.040	<0.000	0.050
**Forced expiratory maneuver**														
BA	2.357 ± 0.905	0.056 ± 0.034	0.029 ± 0.043	0.060 ± 0.039	0.009 ± 0.004	1.555 ± 0.641	0.126 ± 0.261	0.025 ± 0.012	0.019 ± 0.023	0.036 ± 0.092	0.175 ± 0.140	0.019 ± 0.012	0.035 ± 0.044	0.004 ± 0.003
COPD	2.431 ± 1.090	0.060 ± 0.038	0.016 ± 0.017	0.058 ± 0.041	0.012 ± 0.005	1.806 ± 0.733	0.328 ± 0.607	0.034 ± 0.050	0.037 ± 0.029	0.050 ± 0.054	0.129 ± 0.119	0.021 ± 0.012	0.022 ± 0.033	0.003 ± 0.003
Controls	2.107 ± 0.654	0.045 ± 0.020	0.018 ± 0.021	0.079 ± 0.044	0.009 ± 0.003	1.580 ± 0.645	0.061 ± 0.060	0.023 ± 0.012	0.014 ± 0.009	0.032 ± 0.119	0.176 ± 0.124	0.015 ± 0.006	0.030 ± 0.030	0.003 ± 0.003
P value***	<0.000	<0.000	<0.000	<0.000	<0.000	0.009	<0.000	<0.000	<0.000	<0.000	<0.000	<0.000	<0.000	0.031
BA vs. controls	<0.000	<0.000	0.014	<0.000	0.154	0.732	<0.000	0.443	0.055	<0.000	0.290	<0.000	0.936	0.116
COPD vs. controls	<0.000	<0.000	<0.000	<0.000	<0.000	0.009	<0.000	<0.000	<0.000	<0.000	<0.000	<0.000	<0.000	0.186
BA vs. COPD	0.433	0.257	0.055	0.740	<0.000	0.034	<0.000	0.008	<0.000	<0.000	0.001	0.082	<0.000	0.051

Data are presented as mean ± SD or number (%). Data are presented as area comparisons (peak area for the target ion expressed as count per second). *The Kruskale-Wallis test was used for the analysis. **m/z=44.991 presumably corresponds to formic acid-related fragment and m/z=77.059 presumably corresponds to protonated propylene glycol (the putative chemical was identified using Ionicon libraries, the Human Metabolome Database and literature data); ***differences in distributions among all groups. BA: bronchial asthma; COPD: chronic obstructive pulmonary disease; m/z – mass-to-charge ratio; NA: not available; SD: standard deviation.

Table S3. Propensity-Matched Cohort Characteristics

	**Control**	**BA**	**p-value**
N	87	87	
Age, years	57.8 ± 16.3	58.1 ± 16.9	0.884
Sex, male (%)	30 (34.4%)	30 (34.5%)	0.933
Current smokers	9 (10.3%)	10 (11.5%)	0.601
**m/z 79.054**			
Normal quiet breathing	**0.015 ± 0.009**	**0.020 ± 0.022**	**0.018**
Forced expiratory maneuver	**0.014 ± 0.01**	**0.020 ± 0.021**	**0.020**
	**Control**	**BA**	**p-value**
N	87	87	
Age, years	57.8 ± 16.3	58.1 ± 16.9	0.884
Sex, male (%)	30 (34.4%)	30 (34.5%)	0.933
Current smokers	9 (10.3%)	10 (11.5%)	0.601
**m/z 95.054**			
Normal quiet breathing	0.213 ± 0.121	0.196 ± 0.141	0.058
Forced expiratory maneuver	0.212 ± 0.119	0.198 ± 0.139	0.061
	**COPD**	**BA**	
N	69	69	
Age, years	62.3 ± 19.5	63.1 ± 18.9	0.698
Sex, male (%)	32 (46.4%)	32 (46.4%)	1.000
Current smokers	10 (14.4%)	9 (13.0%)	0.845
**m/z 95.054**			
Normal quiet breathing	**0.138 ± 0.133**	**0.189 ± 0.121**	**0.023**
Forced expiratory maneuver	**0.139 ± 0.129**	**0.190 ± 0.119**	**0.021**
	**Control**	**COPD**	**p-value**
N	77	77	
Age, years	65.9 ± 9.8	66.2 ± 10.1	0.841
Sex, male (%)	64 (83.1%)	64 (83.1%)	1.000
Current smokers	12 (15.6%)	13 (16.8%)	0.645
**m/z 118.071**			
Normal quiet breathing	**0.034 ± 0.041**	**0.023 ± 0.033**	**0.022**

Forced expiratory maneuver	**0.030 ± 0.031**	**0.022 ± 0.034**	**0.028**

Data are presented as mean ± SD or number (%). The matching was performed using a nearest-neighbor algorithm with a caliper width of 0.2 of the standard deviation of the logit of the propensity score. Statistically significant p-values ($p < 0.05$) are highlighted in bold. VOC data are presented as normalized peak intensities (counts per second). BDR: bronchodilator responsiveness test; BMI: body mass index.

Table S4. Results of Multivariate Regression Models.

Predictor	**m/z 79.054**			**m/z 95.054**			**m/z 118.071**		
	β (95% CI)	SE	P value	β (95% CI)	SE	P value	β (95% CI)	SE	P value
Diagnosis									
BA	**0.25 (0.11 to 0.39)**	**0.07**	**0.021**	0.12 (-0.02 to 0.26)	0.07	0.089	**0.31 (0.18 to 0.44)**	**0.07**	**0.011**
COPD	**0.19 (0.05 to 0.33)**	**0.07**	**0.038**	-0.09 (-0.23 to 0.05)	0.07	0.204	**-0.22 (-0.35 to -0.09)**	**0.07**	**0.033**
Age, years	-0.01 (-0.03 to 0.01)	0.01	0.254	0.02 (0.00 to 0.04)	0.01	0.047	0.00 (-0.02 to 0.02)	0.01	0.721
Sex (Male)	0.08 (-0.06 to 0.22)	0.07	0.198	0.05 (-0.09 to 0.19)	0.07	0.481	-0.07 (-0.20 to 0.06)	0.07	0.255
Smoking	0.01 (-0.01 to 0.03)	0.01	0.412	0.00 (-0.02 to 0.02)	0.01	0.952	0.01 (-0.01 to 0.03)	0.01	0.387
Model Adj. R^2	0.098			0.065			0.112		

Data are presented as mean ± SD or number (%). β, beta coefficient; CI, confidence interval; SE, standard error. Reference category for Diagnosis: Control group. Reference category for Sex: Female. Statistically significant predictors ($p < 0.05$) are highlighted in bold. β coefficients represent the change in the natural log-transformed VOC peak area per unit change in the predictor.

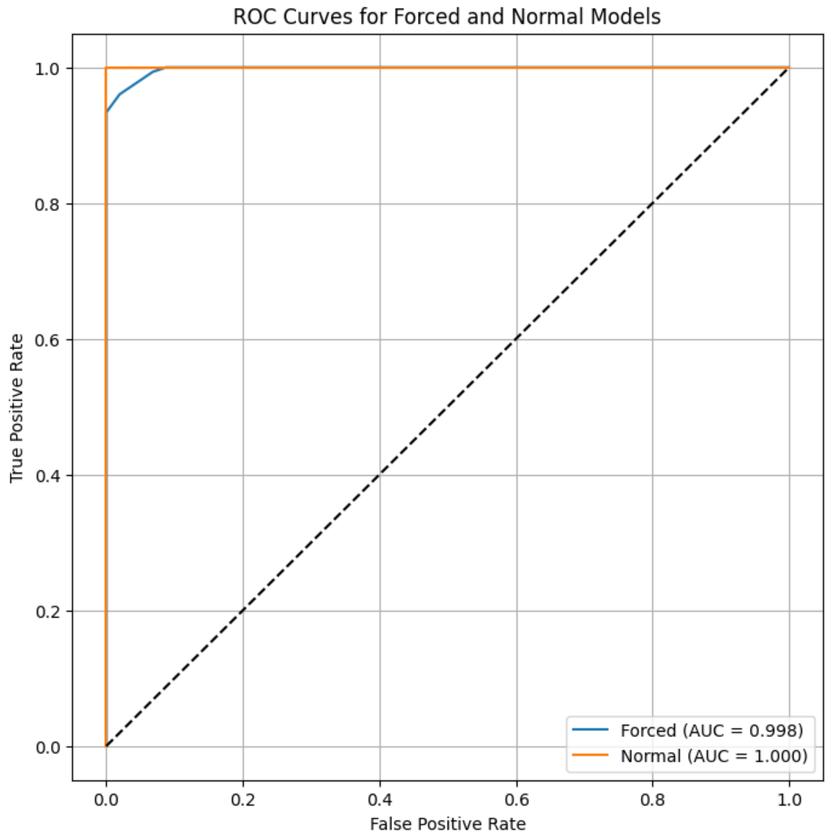

Figure S1. ROC curves for forced and normal expiration models in bronchodilator responsiveness
Forced – ROC curves for forced expiration model; Normal – ROC curves for normal expiration model.

Quality of boosting classifier for BDR with 11 selected VOCs was AUC = 1.000 (sensitivity = 1.000, specificity=1.000) in normal quiet breathing and AUC = 0.970 (sensitivity = 0.960, specificity=0.981) in forced exhale (figure S1). Due to the small amount of data, the models were trained on the entire volume of data, without dividing into training and test sets, in this regard, such a model describes well the patterns in this particular sample.
